# Injecting Patent Blue Dye V for sentinel lymph node biopsy without skin staining

**DOI:** 10.1308/003588412X13171221591259h

**Published:** 2012-05

**Authors:** S Johnson, S Arora, E Babu

**Affiliations:** Hillingdon HospitalLondon, UK

## BACKGROUND

Sentinel lymph node biopsy has become the gold standard technique for staging and avoidance of unnecessary node dissection in breast cancer.^1^ Combinations of blue dye and radioisotope techniques are used to identify the sentinel node. Although accuracy rates are 90- 100%,^2^ injection of blue dye can result in unwanted skin discolouration for up to 18 months.^3^ Although not pathological per se, this can cause ongoing emotional distress to women, serving as a constant reminder of the disease. We describe a simple technique of injecting patent blue dye V so as to minimise skin staining.

## TECHNIQUE

**Figure 1:**
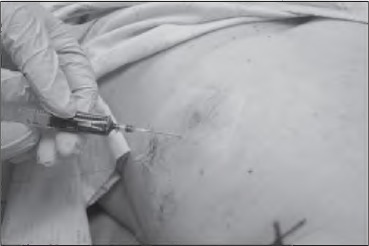
Injection of blue dye with plastic film overlying skin

An adhesive plastic film is laid over the injection site to protect the surrounding skin from any spillage of dye. The dye is then injected subdermally with a 23G needle (Fig 1) and the needle is subsequently withdrawn. Gauze is used to apply 10 seconds of pressure to the injection site to minimise immediate spillage. The plastic film is removed; it will often show minor spillage but this remains on the film as opposed to the patient’s skin.

## DISCUSSION

Injecting blue dye with this technique has been used successfully by us with minimal skin staining in over 100 patients. With sentinel node biopsy being increasingly performed to prevent morbidity associated with axillary clearance,^1^ the potential application of this technique is likely to rise. We hope it will help reduce the number of women with long-term skin staining and thus decrease the psychological consequences of an otherwise very stressful and emotional procedure.
